# Development of persistent HCV genotype 3a infection cell culture model in huh-7 cell

**DOI:** 10.1186/1743-422X-9-11

**Published:** 2012-01-10

**Authors:** Sultan Asad, Bushra Ijaz, Waqar Ahmad, Humera Kausar, Muhammad Tahir Sarwar, Sana Gull, Imran Shahid, Muhammad Kazim Khan, Sajida Hassan

**Affiliations:** 1 Applied and Functional Genomics Lab, Centre of Excellence in Molecular Biology, 87-West Canal Bank Road, 53700 Lahore, Pakistan

**Keywords:** HCV models, siRNA, Huh-7 cell line, HCV Core

## Abstract

**Background:**

Hepatitis C virus (HCV) is one of the major health concerns globally, with genotype 3a as the most prevalent in Pakistan. Lack of efficient HCV genotype 3a small animal models as well as genomic replicons has hampered the complete understanding of its life cycle, pathogenesis and therapeutic options. In this study we aimed to develop a persistent HCV genotype 3a infectious cell culture model.

**Methods:**

We inoculated Huh-7 cells with HCV genotype 3a serum. Cells and media supernatant were collected at different time periods up to 40^th ^day post infection. Culture media supernatant was also collected to find out its ability to infect naive Huh-7 cells.

**Results:**

HCV replication was confirmed at both RNA and protein level through Real Time RCR and western blot using HCV core as marker. In order to validate the persistence of our model for HCV genotype 3a replication we inhibited the HCV replication through core specific siRNAs. The HCV RNA was detected intracellularly from the day one post infection up till 40^th ^day, while HCV core protein was detected from the second day up to 40^th ^day consistently. In culture media supernatant HCV RNA was also actively detected conferring its ability to infect the naive Huh-7 cells. Furthermore, core specific siRNA showed significant inhibition at 24^th ^hour post transfection both at RNA and protein level with progressive increase in the expression of core gene after 3^rd ^day. It clearly depicts that the Huh-7 successfully retained the HCV replication after degradation of siRNA.

**Conclusion:**

Finally, we report that our persistent infection cell culture model consistently replicate HCV genotype 3a for more than 1 month.

## Introduction

HCV is a causal agent of both acute and chronic hepatitis [1] and is one of the foremost health problems affecting nearly 350 million people worldwide [2]. Almost 10% of the population is chronically infected with HCV in Pakistan predominantly by genotype 3a followed by 1a [3-5]. About 40-60% of HCV infected patients lead to chronic liver diseases including liver fibrosis, liver cirrhosis, and hepatocellular carcinoma (HCC) [6,7].

The complete understanding of HCV life cycle and pathogenesis has been impeded due to the unavailability of a competent *in vitro *culture system and appropriate small animal model. Currently, the only well-established immunocompetent animal model for HCV infection is the chimpanzee. However, due to strong ethical concerns, endangered status and high expenses; its wide spread use in hepatitis research has been hampered. Till date, tree shrew (*Tupaia sp.) *is the only small animal model that has been successfully infected by HCV, although only after severe immunosuppression [8]. Immunodeficient urokinase plasminogen activator (uPA) transgenic mice have been used to transplant human hepatocytes, followed by HCV infection [9]. Different HCV transgenic mice have also been produced that express different HCV genes leading to numerous histological changes in mice liver, including development of HCC [10,11]. However, the process of developing small animal model is highly complicated due to the fact that all rodents have to under go xeno-grafting of human liver cells and severe immunosuppression [12].

Different groups have put their efforts to establish cell culture systems highly supportive to HCV replication [13-17]. Although most of the systems permit HCV infection, yet the major draw back is the lack of HCV virions production. However, key progress towards HCV culturing was achieved by the HCV subgenomic replicon development enabling vigorous replication of HCV in culture. In 2005 Wakita et al. successfully cloned HCV genotype 2a JFH1 and transfected it in Huh-7 cell line leading to successful replication and virions production [18], while Zhong et al. achieved a very robust and efficient system for infectious virions in Huh-7 cell line [19]. Yi et al. were able to achieve efficient HCV virions production with HCV genotype 1a J77-S virus in Huh-7 cell line [20]. Despite of their effectiveness the Huh-7 derived HCV virions producing systems have several draw backs like utilization of unusual and rare cloned HCV genotype 2a JFH1 [18] and use of cloned HCV genotype 1a H77-S having five non-natural adaptive mutations [20].

Recently, different groups have studied HCV replication in serum infected liver cell lines and hepatocytes, which mimic the naturally occurring HCV virions biology and kinetics of HCV infection in humans [21-25]. Among them, Huh-7 cell line has been used as model cell culture system to study the mechanisms of HCV associated hepatocarcinogenesis, by using either transient transfection or generating stably transfected cell lines and viral load analysis, as these cells are permissive to HCV infection and replication [25,26].

The subgenomic replicons, small animal models, and infection based cell culture systems are mostly available for genotype 1 and 2; and there is lack of any system particularly for genotype 3a. So, in the current study; we aimed to develop a persistent *in vitro *infection based cell culture model in Huh-7 cells line by infecting them with serum of HCV genotype 3a, providing the cells the environment most closely to the natural one. Furthermore, we evaluated persistent HCV genotype 3a replication in our infection model by silencing HCV genotype 3a replication with siRNA targeting highly conserved core region.

## Results

### Development of persistent HCV genotype 3a huh-7 infectious model

**Figure 1:**
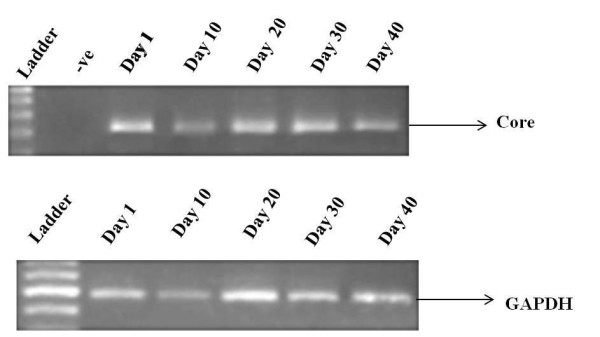
**Expression of core gene at different time periods**. Expression of core gene was detected at different time periods from 1 to 40 days, after infection of Huh-7 cells with HCV genotype 3a serum. Upper row shows continuous core gene expression up to 40^th ^day while the lower row shows GAPDH that was used as internal control.

Huh-7 cells were infected with HCV genotype 3a serum of high titer (> 1 × 10^8 ^copies/μl). RNA was extracted from cells at different days 1, 10, 20, 30 and 40 and reverse transcribed. Detection of HCV RNA was done through HCV genotype 3a core gene specific primers by semi quantitative RT-PCR which showed continuous expression of HCV core gene in serum infected cells at least up to 40^th ^days post infection. GAPDH was used as internal control (Figure 1).

### Monitoring infection of huh-7 cells using culture medium from infected cells by semi quantitative PCR

**Figure 2:**
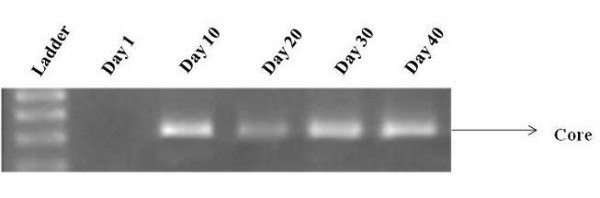
**HCV core gene expressions in media supernatant**. HCV core gene expressions in media supernatant; showed that HCV RNA was detected on the 10^th ^day onward till 40^th ^day post infection. HCV RNA was not detected from 1^st ^to 9^th ^day of post infection.

After incubation of Huh-7 cells with infectious medium presumably containing exocytosed viral particles. The infectious nature of the cells was confirmed by extracting viral RNA from medium. Total RNA from the culture media was extracted at different intervals of time 1, 10, 20, 30, and 40^th ^day post infection and was subjected to semi quantitative RT-PCR with core specific primers. Results showed that viral RNA was coming out of the cells in to culture medium on the 10^th ^day post infection. The expression of core gene in the media suggests that the infected cells exocytose viral particles in the medium that turn naïve cell into infected ones (Figure 2).

### Western blot analysis of HCV core protein expression in serum infected cells

**Figure 3:**
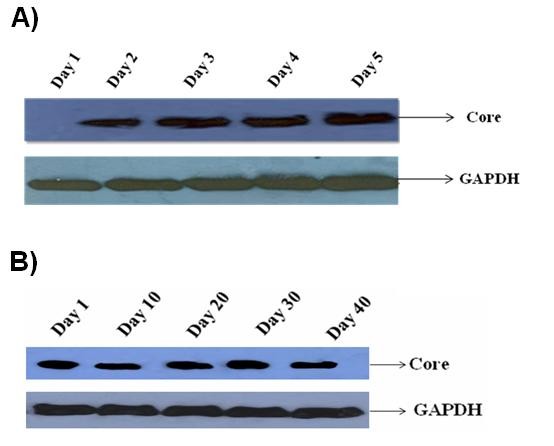
**Confirmation of HCV infection in Huh-7 cells**. (A) The expression level of HCV genotype 3a core protein in serum infected Huh-7 cells through western blot analysis showed gradual increase of HCV core protein from day 1st to day 5th. GAPDH was used as internal control. (B) Continuous expression of HCV Core protein in serum infected Huh-7 cells at different time intervals form 1^st ^to 40^th ^day.

The RT-PCR results were further validated by western blot. The protein was extracted from serum infected cells at different time intervals 1-5, 10, 20, 30, 40^th ^day and was subjected to western blot analysis. Hybridization with anti-core antibody clearly showed the continuous expression of core protein in serum infected cells except at Day 1, possibly due to the fact that there may be lack of development of intermediate negative strand of HCV genotype 3a. GAPDH was used as internal control (Figure 3A, B).

### Effect of core specific siRNA Csi476 on expression of HCV core gene in serum infected huh-7 cells

**Figure 4:**
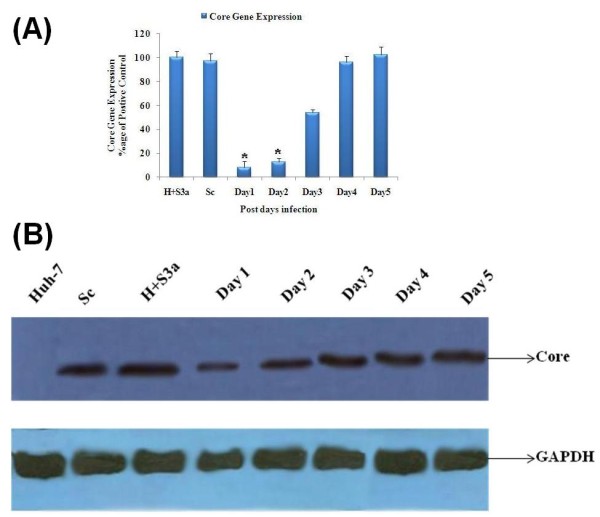
**Inhibitory effect of HCV-3a core gene specific siRNA in Huh-7 infectious model**. (A) Real time Real time PCR of core gene expression level after siRNA (Csi476) transfection in HCV genotype 3a serum infected Huh-7 cells at different days (D1 to D5). Maximum inhibition (92%) was seen at Day 1 of post infection. Core gene expression started to gradually increase till 5^th ^day. Huh-7 cells infected with HCV genotype 3a serum (H + S3a) and scramble siRNA (Sc) used as positive control. Three independent experiments were performed having triplicate samples. Error bars indicate SD, *p < 0.01 GAPDH was used as internal control. (B) Western blot of HCV Core protein in HCV genotype 3a serum infected Huh-7 cells transfected with Csi476 from day one (D1) 1 to day five (D5). Maximum HCV core protein inhibition was seen on day 1 (D1) as compared to Huh-7 cells infected with HCV genotype 3a serum (H + S3a) and scramble siRNA (Sc) that were used as positive control. Huh-7 cells were used as negative control and GAPDH was used as internal control.

We transfected core specific siRNA named Csi476 (100 μM) in our persistent HCV infection model and observed the levels of HCV core gene inhibition at different days (1-5). Results showed significant inhibition of HCV RNA as compared to positive control at 24 h post transfection. HCV replication gradually increased on day 3 and 4 while on 5^th ^day no inhibitory effect of siRNA was seen on serum infected Huh-7 cells. Significant decrease in HCV core gene expression was observed on day 1^st ^(92%, *p < 0.01) and on the day 2^nd ^(88%, *p < 0.01) after transfection (Figure 4A). Western blotting also showed inhibition of the protein expression level of HCV core gene on 1^st ^and 2^nd ^day while GAPDH was used as internal control (Figure 4B).

### Statistical analysis

Statistical analysis was carried out using SPSS software (version 16.0, SPSS Inc). All Data are presented as mean ± SD. Numerical data were analyzed using student's *t*-test. *P *value < 0.05 was considered statistically significant.

## Discussion

To study the pathogenesis and better development of therapy against HCV there is need of a cell culture based system which supports HCV replication. Previously we have used MDBK, HELA, HEK-293 T and Huh-7 cell lines for viral inoculation experiments and found that Huh-7 cell line supported the HCV replication the most [23]. Huh-7 derived cell lines, are most widely used infectious cell culture system for liver associated diseases and fundamental studies for the development of antiviral agents against HCV [19,27,28]. Several alternative strategies are being used to back up the viral component in the different models. Subgenomic replicons system is one of the most commonly used cell culture system to investigate HCV-RNA replication and different steps of viral life cycle [29]. Despite of its effectiveness it can not exactly mimics the actual HCV replication cycle and shedding of viral particles to the medium. In spite of viral replication, the biologically relevant infectious viral particles cannot be demonstrated by such approach. Buck et al. and Molina et al. has successfully infected human primary hepatocytes with the serum of patients infected with HCV genotype 1, 2, 3 and 4 and found efficient HCV replication [21,22].

In the present study, we used serum of HCV genotype 3a infected patient, the most prevalent genotype in Pakistan [3,4] to infect Huh-7 cell line. Recently, it is demonstrated that both 5' and 3' untranslated regions of the viral HCV RNA genome play a pivotal role in translation of viral proteins via interaction with cellular factors including eukaryotic initiation factor 3 eIF3 [30], 40S ribosomal subunit [31], poly pyrimidine tract binding protein (PTB) [32] and microRNA 122 [33]. Besides, it has been shown that intra genetic viral interactions such as NS4a/NS5a are required for key pathways in HCV life cycle. The hypothesis of using HCV infected serum in the present study was, it would have full length HCV genotype 3a RNA genome ensuring the presence of all the necessary ingredients involved in viral replication and poly protein precursor to infect Huh-7 cells *in vitro*. Our results showed the presence of HCV RNA in serum infected cells from day 1 to 40 post serum inoculation. The serum infected cells steadily showed the expression of viral core gene (Figure 1). In the media HCV RNA was not detected till 10^th ^day due to fact Huh-7 cells were not shedding viral particles in to culture medium (Figure 2). This may be due to lack of active exocytosis of viral particles showing absence of replication intermediate [21]. It is interesting to find that Huh-7 showed continuous expression of core protein from 2^nd ^day of infection to 40^th ^day suggesting that replication of HCV is going on in serum infected Huh-7 cells (Figure 3A, B). This finding is in accordance with the earlier work done by El-Awady and his coworkers, who reported HCV genotype 4a serum infection in HepG2 cell line and found that viral proteins started to express themselves 1 week post infection [21]. We presume that our *in vitro *system is highly likely to mimic the *in vivo *HCV replication. Our work is in agreement with the earlier reports of infection experiments [19,23,24].

Furthermore, in this study we evaluated siRNA based HCV genome silencing in our persistent HCV infection Huh-7 cell model that efficiently supported HCV replication up to 40 days. In order to confirm that the HCV is self replicating or due to HCV carry over, we subjected our persistent HCV infection model in Huh-7 cells (post 40 days) to HCV RNA silencing through our previously reported core gene specific siRNA [34]. Excitingly we found significant HCV genotype 3a replication inhibition (*P*-value = 0.00) nearly up to 92% after 24 h (Day 1) post transfection but HCV titer started to increase after 48 h (Day 2) onward and showed continuous replication from 72th hours (Day 3) (Figure 4A), whereas the control siRNA (scramble) did not show any effect on inhibition of HCV replication. HCV replication in the Huh-7 cells was observed through semi quantitative RT-PCR by using core specific primers. Western blot results also showed significant down regulation of core protein at day one of post transfection (Figure 4B). These results validate our previous study in which we found significant inhibition in transiently infected Huh-7 cells. Our data is in disagreement with Zekri et al. [26] who demonstrated that siRNAs against 5'UTR of HCV genotype-4 inhibited HCV replication in serum infected Huh-7 cells up to 7 days. Our results clearly depict that the most epic inhibitory effect of siRNA was seen 24 h post transfection. The difference may be due to the selection of two different HCV regions and genotype.

## Conclusions

We report that our infectious cell culture model in Huh-7 cell line persistently support HCV genotype 3a replication *in vitro*. The continuous expression of HCV proteins and capacity of culture medium to transmit the virus to naïve cells depicts this model as an efficient one to evaluate HCV therapeutic options and molecular studies.

## Materials and methods

### Serum sample collection

The local HCV genotype 3a patient's serum samples were obtained from the CAMB diagnostic laboratory, Lahore, Pakistan. Serum samples were stored at -70°C prior to RNA extraction. For viral inoculation experiments, 1 × 10^8 ^IU/ml viral load of genotype 3a was used. HCV genotypes were determined by CAMB diagnostic laboratory, Lahore, Pakistan. The study was approved by institutional ethics committee and Patient's written consent was obtained.

### Cell culturing

Huh-7 cell line was maintained in Dulbecco's modified eagle medium (DMEM) supplemented with 100 μg/ml penicillin; streptomycin and 10% fetal bovine serum referred as complete medium (Sigma Aldrich, USA) at 37°C with 5% CO_2_. The medium was renewed every 3^rd ^day and passaged every 4-5 days. Viable cells were counted using 0.5% trypan blue (Sigma Aldrich, USA). For 6-well plates briefly 3 × 10^5^cells/well were plated and cultured in complete medium until 60-80% confluent.

### Viral inoculation experiment

For viral inoculation, we used similar protocols as established by Khaliq and co-workers (2010) with slight modifications. High viral titer > 1 × 10^8 ^IU/ml from HCV genotype 3a patients was used as principle inoculums in these experiments. Briefly, 3 × 10^7 ^Huh-7 cells were seeded in 60 mm culture plates, in DMEM (Sigma Aldrich, USA) as described under cell culturing heading. On semi-confluency, cells were washed twice with serum-free medium, then inoculated with 500 μl (1 × 10^8 ^IU/well) of HCV genotype 3a serum and 500 μl serum free media. After 3 h media was diluted such that the serum concentration becomes 10%. Cells were maintained overnight at 37°C in 5% CO_2_. The next day, adherent cells were washed three times with 1X PBS, and the incubation was continued in CCM.

### siRNA transfection

siRNA used in this study named Csi476 was raised against highly conserved C terminal sequence of HCV core gene. Csi476 antisense **AAGACGGGATAAATTTCGCAACCTGTCTC **and Csi476 sense **AATTGCGAAATTTATCCCGTCCCTGTCTC**. Scramble siRNA used in this study was termed sc and its sequence was antisense **AACCTGCATACGCGACTCGACCCTGTCTC **and sense strand **AAGTCGAGTCGCGTATGCAGGCCTGTCTC**.

To analyze the effect of siRNA on HCV infection, serum infected Huh-7 cells were seeded in 6-well (3 × 10^5^/well) plates and cultured in CCM until they became 60-80% confluent. Transfection was performed with 100 μM/well core specific siRNA Csi476 or scrambled siRNA in serum free media using Lipofectamine™ 2000 (Invitrogen) according to the manufacturer's protocol.

### Total RNA isolation

Total RNA was extracted from the media, HCV infected Huh-7 cells or siRNA transfected HCV infected Huh-7 cells at different time periods using the Pure script^® ^RNA Isolation kit (Gentra, USA) according to manufacturer protocol. RNA samples were stored at -70 to -80°C until use or otherwise proceeded toward cDNA synthesis.

### Detection of HCV RNA through semi quantitative RT-PCR

Total RNA (1 μg) extracted was subjected to reverse transcription with the help of oligo dT primer. Then the cDNA was subjected to PCR with the Core gene specific primers (Forward **CCGTTGGCATGAAGTGTATG **and Reverse **CCAGTGAAGAGAGCCTGACC)**. GAPDH was used as internal control (Forward Primer **ACCACAGTCCATGCCATCA and **Reverse Primer **TCCACCACCCTGTTGCTGTA)**. The PCR products were subjected to electrophoresis to find out the desired bands of HCV Core gene or GAPDH.

### Quantification of core gene expression using real time PCR

The effect of siRNA on HCV replication was analyzed by expression analysis of HCV Core gene using specific-primers of HCV Core on ABI 7500 Real Time PCR using SYBR Green mix (Fermentas) according to manufacturer's instructions. Template cDNA 0.5 μg was used for the quantification of expression. GAPDH gene was used as an internal control for normalization. The relative gene expression analysis was done by using SDS 3.1 software provided by ABI. Each Real Time PCR assay was performed in triplicate. Level of Significance and standard error were determined by SPSS software for Windows.

### Western blotting

To determine the protein expression levels of HCV Core, in serum infected, and siRNA transfected (with and without HCV genotype 3a Core siRNA Csi476 and scramble siRNA) and non-transfected cells were lysed with ProteoJET mammalian cell lysis reagent (Fermentas, Canada). Equal amounts of total protein were subjected to electrophoresis on 12% SDS-PAGE and electrophoreticallty transferred to a nitrocellulose membrane following the manufacturer's protocol (Bio-Rad, CA). After blocking non-specific binding sites with 5% skimmed milk, blots were incubated with primary monoclonal antibodies specific to HCV Core and GAPDH (Santa Cruz Biotechnology Inc, USA) and secondary Horseradish peroxidase-conjugated anti-goat anti-mouse antibody (Sigma Aldrich, USA). The protein expressions were evaluated using chemiluminescence's detection kit (Sigma Aldrich, USA).

## Abbreviations

HCV: Hepatitis C virus; DMEM: Dulbecco's modified eagle medium; HCC: Hepatocellular carcinoma; siRNA: small interfering RNA; CCM: complete culture medium; MDBK: Madin-Darby bovine kidney; HELA: Human epithelial carcinoma; HEK: Human embryonic kidney; CAMB: Centre of applied molecular biology.

## Competing interests

The authors declare that they have no competing interests.

## Authors' contributions

SA, BI and WA contributed equally to this work. SA, BI and WA analyze the data and helped in paper write up. MTS, SG, HK, IS and MKK maintained the cell cultures and carried out the cell culture experiments. SH designed the study; also checked the revised manuscript thoroughly and confirmed all the data given in manuscript. All work was performed under supervision of SH. We all authors read and approved the final manuscript.

## Authors' information

SA, BI, HK, and MTS are Ph.D. scholars in discipline of Molecular Biology at CEMB, University of the Punjab, Lahore, WA (M Phil Chemistry) and SG (MSc Biochemistry) are Research Officers, MKK is M. Phil scholar; while SH (PhD Molecular Biology) is Principal Investigator at CEMB, University of the Punjab, Lahore.
